# Concealed Peritonitis Due to Stomach Perforation from a Fish Otolith: A Case Report

**DOI:** 10.3390/reports8040252

**Published:** 2025-11-28

**Authors:** Matthew E. Falagas, Laura T. Romanos, Annabel Hopkins, Athanasios Sioulas, Maria Tsitskari

**Affiliations:** 1 Alfa Institute of Biomedical Sciences (AIBS), 151 23 Marousi, Athens, Greece; l.romanos@aibs.gr; 2 School of Medicine, European University Cyprus, 2404 Nicosia, Cyprus; m.tsitskari@euc.ac.cy; 3 Department of Medicine, Tufts University School of Medicine, Boston, MA 02111, USA; 4 Department of Imaging, Hygeia Hospital, 151 23 Athens, Greece; abhopkin@gmail.com; 5 Department of Medicine, Hygeia Hospital, 151 23 Athens, Greece; athsioulas@yahoo.gr

**Keywords:** ampicillin/sulbactam, foreign body, gastrointestinal tract, intra-abdominal infection, perforation, pylorus

## Abstract

**Background and Clinical Significance:** Ingestion of foreign bodies may lead to perforation of the gastrointestinal tract in its various segments. This may be accompanied by infections of the mediastinum after esophageal perforations and peritonitis after perforations of the stomach and bowel. **Case Presentation:** A 64-year-old man was admitted to the hospital because of abdominal pain and fever. The laboratory testing showed increased indices of inflammation. A CT scan of the abdomen revealed perforation of the stomach pylorus wall from a foreign body. Additionally, there were imaging findings suggesting concealed peritonitis in the adjacent area of stomach perforation. A 3.9 cm foreign body was removed with gastroscopy. The investigation into the nature of the foreign body suggested that it was a fish otolith (a structure composed of calcium carbonate, also known as an ear bone). The patient adhered to a Mediterranean diet. He recalled ingesting parts of the head of a 2.5 kg sea bream about 40 days before his admission to the hospital. The patient received broad-spectrum antimicrobial treatment, specifically intravenous ampicillin/sulbactam (2 g/1 g) every 8 h. He had complete resolution of his infection, with full resolution of symptoms and normalization of all abnormal signs noted in the physical examination at outpatient follow-up. **Conclusions:** Ingestion of a fish otolith may lead to perforation of the gastrointestinal tract and subsequent intra-abdominal infection. Prompt diagnosis with abdominal imaging, especially a CT scan, removal of the foreign body by upper gastrointestinal endoscopy (if possible), and broad-spectrum antibiotics are necessary for the successful management of such cases.

## 1. Introduction

Ingestion of foreign bodies may lead to perforation of the gastrointestinal tract at various segments, including the esophagus, stomach, small intestine, and large intestine [1,2,3,4,5,6]. The perforation of the gastrointestinal tract at the level of the esophagus may lead to mediastinal infections. Such infections in the mediastinum may be severe with devastating consequences [7,8,9,10]. Perforation of the gastrointestinal tract due to ingestion of a foreign body of various types, including bones, metals, and magnets, may have serious consequences for the patient, especially if there is a delay in the diagnosis and/or inappropriate use of antimicrobial treatment. In this article, we present a case of perforation of the stomach in the pylorus by a foreign body, leading to concealed peritonitis [11,12,13,14,15,16,17,18,19,20,21,22].

## 2. Case Report

A 64-year-old male patient was admitted to the hospital via the emergency department due to severe abdominal pain, fever, and malaise. His symptoms started about two days before his admission to the hospital and had increasing intensity. He did not have any diarrhea or changes in the color of his stool.

His past medical history was positive for arterial hypertension. He had no previous history of gastrointestinal tract disease, neoplasia, diabetes mellitus, aortic or other vascular disease. His body weight was normal.

The physical examination revealed tenderness in the epigastric area. There was no rebound tenderness or abdominal rigidity. His bowel sounds were normal. The rest of the physical examination, including the respiratory, cardiovascular, and urinary systems, revealed no abnormal findings.

The laboratory testing revealed an elevated white blood cell count (15.940/mm^3^ of blood) with neutrophilia (polymorphonuclear white blood cells 89%). Also, there was an elevation of C-reactive protein (CRP) [21.01 mg/dL (normal range: ≤0.5 mg/dL)] and D-dimer (2.11 µg/mL (normal range: ≤0.5 µg/mL)). The rest of the routine hematological and biochemical testing, including hemoglobin, hematocrit, platelet count, transaminases, cholestatic enzymes, amylase, direct and indirect bilirubin, urea, creatinine, and electrolytes, were all within normal ranges.

**Figure 1:**
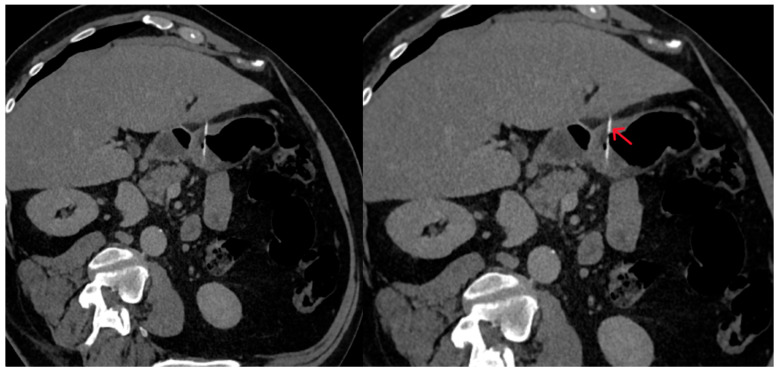
The foreign body is observed within the stomach lumen, projecting outside the stomach wall margins, suggesting perforation (red arrow).

**Figure 2:**
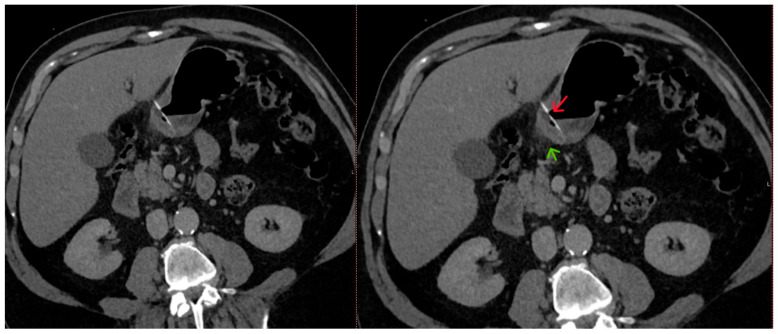
A linear foreign body is present in the stomach lumen (red arrow), perforating the gastric wall, with surrounding fat stranding (green arrow) resulting from inflammation.

**Figure 3:**
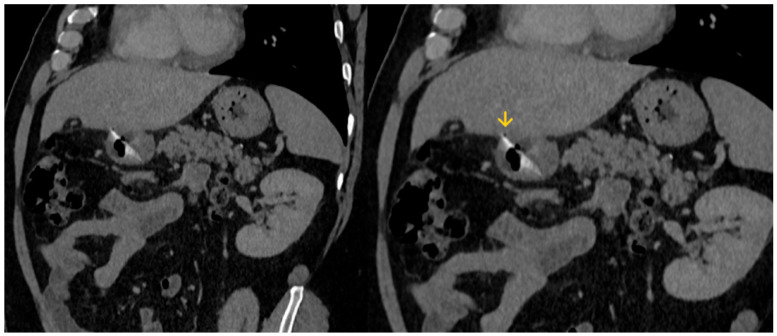
A small amount of gas (yellow arrow) is depicted outside the stomach wall, due to perforation.

The abdominal CT scan showed a foreign body in the pylorus of the stomach that was penetrating the wall of the stomach (Figure 1). There was also air outside the stomach, along with signs of inflammation in the adjacent adipose fatty tissue (Figure 2 and Figure 3). The diagnosis of perforation of the stomach wall with a foreign body was made, leading to subsequent concealed, local peritonitis.

The patient was administered intravenous antimicrobial treatment with ampicillin/sulbactam (2 g/1 g) every 8 h. Ringer’s lactate intravenous hydration (80 mL/hour), and intravenous omeprazole. Additionally, he did not receive food or water orally for 6 days.

**Figure 4:**
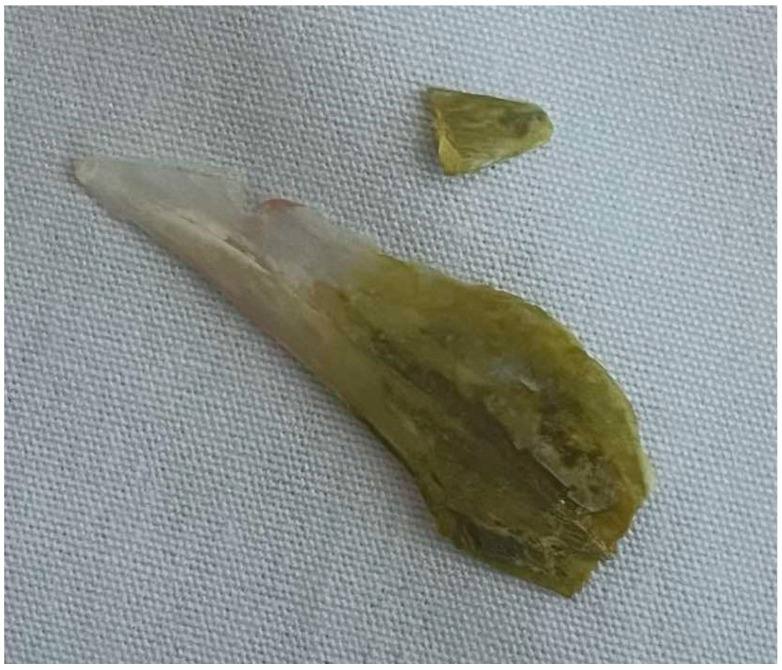
The removed foreign body was a fish otolith (part of it had a greenish appearance).

**Figure 5:**
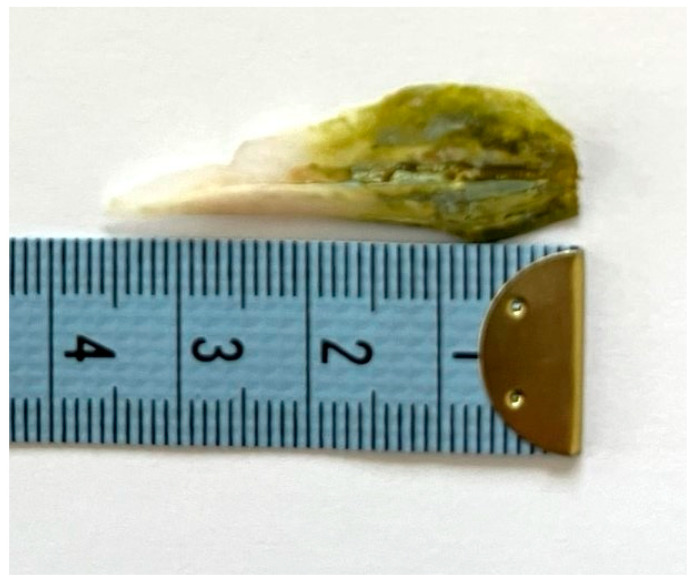
The length of the removed foreign body was about 3,9 cm (together with the small broken piece of it shown in Figure 4).

A gastroscopy was performed two days after his admission to the hospital for the removal of the foreign body. A 3.9 cm body was removed that looked like a fish bone (Figure 4 and Figure 5).

The patient continued to experience abdominal tenderness in the epigastric area due to ongoing local inflammation (concealed peritonitis). There was a gradual reduction in CRP [13.02 mg/dL on the third day, 4.63 mg/dL on the fifth day, 1.93 mg/dL on the seventh day, 0.90 mg/dL on the ninth day of his hospitalization]. D-dimer was still elevated on the ninth day of his hospitalization (1.16 µg/mL).

Due to the severity of his overall clinical condition, the patient continued to receive intravenous antimicrobial treatment [intravenous ampicillin/sulbactam (2 g/1 g) every 8 h] and intravenous fluids for an additional three days. He was discharged after 11 days of hospitalization and had medical follow-up on an outpatient basis. The patient received outpatient antimicrobial treatment (moxifloxacin 400 mg tablet once a day) for six days. His health returned to normal, with no symptoms, signs from the physical examination, or abnormalities in his hematological and biochemical testing, including indices of inflammation.

## 3. Discussion

Perforation of the gastrointestinal tract is a rare complication after ingestion of foreign bodies [10,23,24,25,26,27,28,29,30]. Overall, it occurs in about 1% of foreign body ingestions. However, the probability of perforation of the gastrointestinal tract greatly depends on the specific characteristics of the ingested foreign body. Most reported perforations are caused by sharp, pointed objects (such as chicken bones, fish bones, toothpicks, metallic staples, and metallic wires) rather than smooth, small calcified structures. The perforation of the gastrointestinal tract usually occurs soon after the ingestion of the foreign body (within two weeks). However, there are case reports of unusual delayed perforations related to various ingested foreign bodies [31,32]. For example, a wooden chopstick ingestion was associated with duodenal perforation 9 months later. The patient in that case report was treated with laparotomy, foreign body removal, and duodenography [33].

The clinical manifestations after perforation of the gastrointestinal tract by foreign bodies may be unusual and sometimes surprising to clinicians. For example, the perforation of the large intestine by a fish bone led to left lower quadrant abdominal pain with increasing intensity for 12 days in an 80-year-old male. The clinical presentation and findings from the imaging studies on that patient led to the excision of a mass that was adjacent to the sigmoid colon and had signs of an abscess. A sharp fish bone was found intraoperatively, although no relevant findings were noted preoperatively [34].

The investigation of the nature of the removed foreign body in our case suggested that it was a fish otolith. Otoliths are also known as ear stones or ear bones. In humans, otoliths are microscopic, are made of calcium carbonate embedded in a gel-like matrix, and are located inside the vestibular system of the inner ear (utricle and saccule). They play a crucial role in maintaining balance and detecting gravity. In fish, otoliths are larger and more mineralized than in mammals. They play a role in detecting sound, balance, and movement. Fish otoliths are located in the inner ear and grow in layers (like tree rings). Subsequently, they serve as “biological black boxes” because scientists can measure their rings to determine a fish’s age and track growth [35]. Fisheries biologists extract otoliths to study fish age, population dynamics, and migration patterns [36]. Additionally, the chemical composition of otoliths can reflect the water chemistry where the fish lived.

The patient in our case adhered to a Mediterranean diet and frequently consumed various species of fish. He recalled eating parts of the head of a large sea bream weighing approximately 2.5 kg about 40 days before his hospital admission. Such a fish measures approximately 60 cm in length and may have long otoliths. Additionally, the patient recalled that he had vague complaints of dyspepsia during the last month before his hospital admission.

The greenish color of the part of the fish otolith removed from the stomach of our patient could be attributed to the staining with bile, regurgitated from the duodenum to the stomach pylorus. Additionally, bacterial infections can alter the color of the surface of foreign bodies. For example, *Pseudomonas aeruginosa* may lead to the production of greenish pus and the formation of greenish biofilms due to the excretion of color-producing substances, including pyoverdine and pyocyanin.

The frequent development of abdominal infection after perforation of the stomach or the bowel by an ingested foreign body necessitates the administration of antimicrobial treatment. The severity of the condition (concealed or non-concealed peritonitis) leads to inpatient management, including intravenous antibiotics. The main differential diagnosis of severe epigastric pain, which was one of the symptoms of our patient, includes peptic ulcer disease, severe gastritis, perforation of the stomach or the duodenum, pancreatitis, biliary tree disease, aortic dissection, and inferior wall myocardial ischemia. Perforation of the ascending colon may lead to pain in the right lower abdominal quadrant. Sometimes it is accompanied by minimal signs in the physical examination. Differential diagnosis of right lower quadrant pain with minimal physical findings includes acute appendicitis (in the case of a retrocecal appendix, since this location prevents direct peritoneal irritation). It also includes acute acalculous cholecystitis (especially among elderly patients who may have only minimal findings). Another condition to consider is a perforated peptic ulcer as microperforations may seal quickly. Although such conditions usually cause epigastric pain, this pain may be referred to the right lower quadrant as the fluid descends. Early bowel obstruction should also be considered, as the initial stages may present with mild, nonspecific symptoms. Infections resulting from perforation of the gastrointestinal tract typically have a mixed microbial etiology, comprising both aerobic and anaerobic Gram-negative and Gram-positive bacteria. Thus, combinations of antibiotics or drugs with a broad antimicrobial spectrum are used for this indication [37,38,39,40].

Concealed peritonitis may occur in patients with various types of intra-abdominal infections, including appendicitis and diverticulitis. In some cases, symptoms may be limited and mild. Additionally, patients with concealed peritonitis may not have apparent signs from the physical examination. Thus, the clinical condition of a patient with concealed peritonitis may be underestimated, leading to delay of appropriate diagnostic and therapeutic measures.

Fluoroquinolones and β-lactam antibiotics are among the first-line choices for patients with abdominal infection due to perforation of the gastrointestinal tract by an ingested foreign body, similarly to cases with intrabdominal infections of other causes [41,42,43,44,45,46,47,48,49,50]. A β-lactam/β-lactamase inhibitor, specifically ampicillin/sulbactam, was selected as the antimicrobial treatment in our patient. Additionally, combinations of antimicrobial agents, including metronidazole due to its excellent activity against anaerobic bacteria, such as *Bacteroides fragilis* and other Bacteroides species, are frequently considered in patients with abdominal infection following perforation of the stomach or bowel by an ingested foreign body [51,52,53,54,55,56].

Our case report highlights the significance of clinical awareness of the possibility of perforation of the gastrointestinal tract by a foreign body in patients with symptoms of abdominal infection. It is also didactic for gastrointestinal endoscopists and surgeons because it presents a case of perforation of the stomach that led to clinical manifestations late after ingestion of the foreign body. Surgical management may be necessary in cases of generalized peritonitis. Our case also underscores that localized peritonitis is a severe infection with potentially devastating consequences. The considerably increased CRP reflected the severity of the abdominal infection in our patient with concealed peritonitis after stomach perforation caused by the ingested foreign body.

## 4. Conclusions

In our case, the patient developed concealed peritonitis after perforation of the stomach pylorus wall from a fish otolith. Prompt diagnosis, especially using CT scan imaging, an effort to remove the foreign body by gastroscopy if technically feasible, along with the administration of broad-spectrum antimicrobial treatment, are necessary components for a successful outcome.

## Data Availability

The original contributions presented in this study are included in the article. Further inquiries can be directed to the corresponding author.
